# Leveraging Citizen Science and Low-Cost Sensors to Characterize Air Pollution Exposure of Disadvantaged Communities in Southern California

**DOI:** 10.3390/ijerph19148777

**Published:** 2022-07-19

**Authors:** Tianjun Lu, Yisi Liu, Armando Garcia, Meng Wang, Yang Li, German Bravo-villasenor, Kimberly Campos, Jia Xu, Bin Han

**Affiliations:** 1 Department of Earth Science and Geography, California State University, Dominguez Hills, Carson, CA 90747, USA; agarcia499@toromail.csudh.edu (A.G.); gbravovillasenor1@toromail.csudh.edu (G.B.-v.); kcampos44@toromail.csudh.edu (K.C.); 2 Department of Population and Public Health Sciences, Keck School of Medicine, University of Southern California, Los Angeles, CA 90089, USA; yisil@usc.edu; 3 Department of Epidemiology and Environmental Health, School of Public and Health Professions, University at Buffalo, Buffalo, NY 14214, USA; mwang54@buffalo.edu; 4 Department of Environmental Science, Baylor University, Waco, TX 76798, USA; yang_li3@baylor.edu; 5 State Key Laboratory of Environmental Criteria and Risk Assessment, Chinese Research Academy of Environmental Sciences, Beijing 100012, China; carol3233@126.com (J.X.); hanbin@craes.org.cn (B.H.)

**Keywords:** exposure assessment, low-cost sensing, public engagement, traffic-related air pollution, environmental inequality

## Abstract

Assessing exposure to fine particulate matter (PM_2.5_) across disadvantaged communities is understudied, and the air monitoring network is inadequate. We leveraged emerging low-cost sensors (PurpleAir) and engaged community residents to develop a community-based monitoring program across disadvantaged communities (high proportions of low-income and minority populations) in Southern California. We recruited 22 households from 8 communities to measure residential outdoor PM_2.5_ concentrations from June 2021 to December 2021. We identified the spatial and temporal patterns of PM_2.5_ measurements as well as the relationship between the total PM_2.5_ measurements and diesel PM emissions. We found that communities with a higher percentage of Hispanic and African American population and higher rates of unemployment, poverty, and housing burden were exposed to higher PM_2.5_ concentrations. The average PM_2.5_ concentrations in winter (25.8 µg/m^3^) were much higher compared with the summer concentrations (12.4 µg/m^3^). We also identified valuable hour-of-day and day-of-week patterns among disadvantaged communities. Our results suggest that the built environment can be targeted to reduce the exposure disparity. Integrating low-cost sensors into a citizen-science-based air monitoring program has promising applications to resolve monitoring disparity and capture “hotspots” to inform emission control and urban planning policies, thus improving exposure assessment and promoting environmental justice.

## 1. Introduction

Ambient (outdoor) air pollution (e.g., fine particulate matter with a diameter less than 2.5 μm (PM_2.5_)) is estimated to cause 4.6 million premature deaths annually worldwide [1] and is widely associated with various adverse health effects including cardiovascular and respiratory diseases [2,3]. For decades, evidence has suggested that communities of low income, low education attainment, and people of color (often referred to as disadvantaged communities) are more likely to live near freeways and industrial facilities and are exposed to higher levels of air pollution, leading to environmental health disparities [4,5,6]. For example, a recent national-level study in the US found that racial/ethnic minority populations are more than two times as likely to expose to higher air pollution levels than non-Hispanic White populations [7]. In terms of source contributions to such racial/ethnic disparity in PM_2.5_ exposure, traffic-related sources (e.g., heavy-duty diesel vehicles) are among the top four source sectors [8]. Studies also found that the impacts of PM_2.5_ on life expectancy were especially pronounced among people of color populations [9]. The disparities in exposure to air pollution warrant further studies on exposure assessment across disadvantaged communities.

Over the years, the US Environmental Protection Agency (US EPA) has been actively monitoring ambient air quality through the largest national air monitoring network worldwide, but it is insufficient to cover a variety of areas due to cost and power constraints [10]. For example, out of 3100 counties in the US, only 21% have PM_2.5_ monitors, of which ~48% have only one monitor [11]. Additionally, these regulatory monitors are generally optimized to provide the “gold standard” for regulation compliance by tracking regional-scale trends in air quality across large geographical areas [12]. The lack of spatial coverage and density may not adequately reflect community-level variations and disparities [13], thus leading to biases in the study of epidemiology, urban planning, and environmental justice [14,15].

Emerging low-cost sensing technologies have the advantage of increasing the spatial resolution of air pollution measurements and supplementing regulatory data with a much lower cost [16,17]. The use of these accessible low-cost sensors has also democratized air pollution data collection by shifting air quality monitoring from government agencies towards citizen science, crowd-sourced, or community-based approaches [18,19,20,21]. Recent studies found that citizen-science-based low-cost sensing data could be leveraged to target more locations of interest and identify air pollution “hotspots” [22,23,24]. Citizen science also allows for co-creation through citizen involvement in the scientific process and results in greater data accessibility and better knowledge production [18,25]. However, the rise of low-cost monitoring strategies may reveal exposure assessment inequality [26]; that is, low-cost sensors are installed at locations that are less representative in terms of concentration levels and sociodemographic characteristics [10]. For example, as one of the largest public low-cost sensing networks, PurpleAir sensors are more likely to be deployed in communities with White, richer, and more educated residents [10]. Questions remain on how citizen science could be combined with low-cost sensing to resolve the lack of air monitoring data across disadvantaged communities.

Additionally, while there are uncertainties from the low-cost sensors related to data sensitivity to temperature, relative humidity, as well as particle coincidence, many recent studies have successfully applied careful field calibrations with reference measurements to reduce such biases and achieved reliable performance. Particularly, a national correction equation of PurpleAir sensors has been recently developed by the US EPA to improve the feasibility and practicability of such sensor networks [27]. Overall, it is significant to resolve the gap in air monitoring to improve air pollution exposure through citizen science and low-cost sensing, particularly for disadvantaged communities with limited resources and attention.

Southern California holds counties (e.g., Los Angeles County (LA County)) in the US with the highest annual PM_2.5_ levels, the largest port complex (Port of LA), and a vast network of freeways and industrial facilities. It is estimated that traffic-related air pollution contributes to 45% of the PM_2.5_ in the region [28,29]. Southern California also consists of many disadvantaged communities that are in proximity to major freeways and disproportionately burdened by poor air quality [30]. In terms of exposure assessment, a recent study in LA County identified societal gaps in access to air pollution exposures of low-cost sensors among disadvantaged communities, suggesting the need to expand the density of monitoring networks through citizen science while advancing environmental justice efforts [31]. Additionally, little guidance is offered on how to better resolve such air pollution exposure disparities, and few studies have discussed potential strategies to improve air quality.

In this study, we applied citizen science by engaging 22 households from 8 communities in Southern California to launch a low-cost air pollution sensor network (i.e., community air monitoring program (CAMP)). The monitoring network tracked PM_2.5_ levels for at least two weeks consecutively during both summer and winter 2021. We focused on (1) identifying the spatial and temporal patterns of the PM_2.5_ exposure and (2) understanding the contribution of diesel emissions to ambient PM_2.5_ concentrations. Our work is one of the few studies that evaluate air pollution exposure disparities based on newly launched monitoring programs across disadvantaged communities. Our study may shed light on how to translate the exposure assessment into actionable policies to promote environmental justice for disadvantaged communities that are heavily burdened by various emission sources.

## 2. Materials and Methods

### 2.1. Study Area

**Figure 1:**
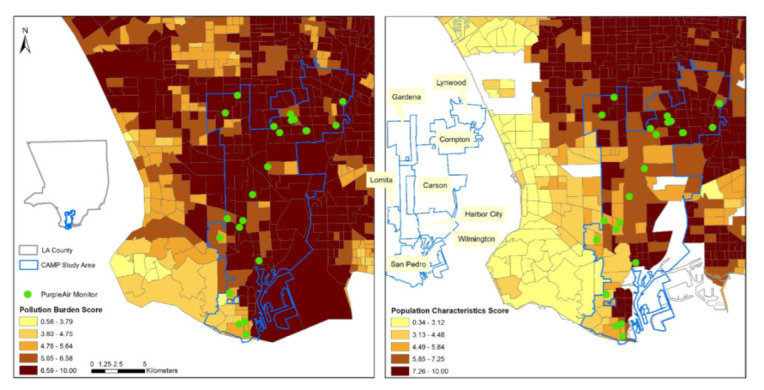
CAMP study area with the pollution burden and population characteristics.

Our CAMP study area is located in LA County, a large, populous area in Southern California (~10 million population in 2343 census tracts) with a wide spectrum of socioeconomic disparities across communities [32]. According to the US EPA, among the 229 metropolitan areas, LA is one of the most polluted regions in the US [33]. The CAMP study area includes neighborhoods from 11 communities and unincorporated areas (hereafter, communities). This area (215 km^2^) consists of 131 census tracts with ~630,000 population around California State University, Dominguez Hills (CSUDH; located in Carson, CA and is one of the largest Hispanic Serving Institutions in the US). Most of the CAMP study area are disadvantaged communities with low income (70%) and high proportions of minorities (56% Hispanic, 14% African American, and 13% Asian American) and susceptible populations (13% aged above 65 years old and 13% below 10 years old) with disproportionate environmental pollution burdens [32]. The area is also surrounded by several major interstate and state highways (e.g., State Route 91, Interstate 710, Interstate 110, and Interstate 105). Figure 1 shows the CAMP study area with the pollution burden and population characteristics.

### 2.2. Participant Recruitment

Our CAMP study team reached out to university students, faculty, alumni, and connected organizations via email to recruit resident participants from the 11 targeted communities; a total of 66 residents responded with strong interest. These residents were further selected to balance the number of selected residents in each community, neighboring transportation facilities and land uses, and distance among each household. This process resulted in 22 participants from 8 communities (i.e., Carson, Compton, Gardena, Harbor City, Lomita, Lynwood, San Pedro, and Wilmington), and about 85% were from disadvantaged communities according to the CalEnviroScreen 4.0, a tool to identify California communities that are disproportionately burdened by various sources of pollution [32]. Our study received the CSUDH Institutional Review Board approval (20-255) on 29 April 2021.

### 2.3. Monitoring Campaign

As one of the most popular and reliable low-cost sensor networks, PurpleAir sensors have been commonly used by universities, industrial organizations, and government air districts to effectively measure community-level air pollution using a light-scattering technique (www2.purpleair.com). One PurpleAir low-cost sensor unit was installed outside of each participant’s home (e.g., backyard, porch); each household was monitored for at least two weeks to characterize exposures for weekdays and weekends in both the summer (2 June to 27 August) and winter of 2021 (29 October to 13 December 2021). The primary goal was to track PM_2.5_ exposure across different time periods and households among disadvantaged communities.

### 2.4. Quality Assurance and Quality Control (QA/QC)

Various studies have found that PurpleAir sensors were well correlated with regulatory-grade instruments [34,35] and could be carefully calibrated to offer valuable exposure information [24,27,36]. We applied a rigorous QA/QC procedure to process the raw measurements from the CAMP study. Each PurpleAir sensor includes two sensor channels, but studies suggested that both channels generally agreed well [24,27]. Therefore, we primarily used measurements from Channel A. While these types of low-cost sensors are sensitive to relative humidity, temperature, and particle coincidence [37], a recently developed national PurpleAir correction equation found that a correction equation with an additive relative humidity term showed the best performance [27]. Therefore, our correction equation included a relative humidity term. Since the national correction equation was created based on collocated monitoring data across diverse regions in 16 states in the US, we instead applied a local hourly correction equation by using a PurpleAir sensor collocated with a regulatory-grade monitor (ID: 060371302) of South Coast Air Quality Management District (South Coast AQMD) in the City of Compton, one of the communities within our study area; the data from January to June 2021 were extracted considering data availability. The raw PurpleAir 2 min measurements were aggregated into hourly data. Then, we used the local hourly correction equation to adjust all hourly measurements of the CAMP study.

### 2.5. Data Analysis

We analyzed the CAMP PM_2.5_ measurements of the 22 households among disadvantaged communities to identify: (1) the air pollution exposure across space and time and (2) the relationship between the total PM_2.5_ measurements and diesel PM emissions. The overarching goal was to evaluate how residents from disadvantaged communities were exposed to ambient air pollution and how measures could be taken to relieve the pollution exposure.

To explore how PM_2.5_ exposure varied across communities with different built-environment and socioeconomic conditions, we calculated the community-level average PM_2.5_ concentrations during all monitoring days. We also compared the community-level average concentrations to the major indicators assembled from the CalEnviroScreen 4.0, namely traffic density, poverty, unemployment, housing-burdened low-income households, population characteristics, and percent of racial/ethnic population in the census tract level. Table S1 shows the detailed description of all assembled variables. To identify how PM_2.5_ exposure changed across different time periods, we summarized the calibrated measurements based on multiple time periods, including the hour of the day, the hour of the day by season, the hour of the day by weekday vs. weekends, and day of the week.

While evaluating the spatial and temporal patterns of air pollution exposure is significant for disadvantaged communities, exploring measures that could be implemented to reduce the exposure is also needed, especially considering that these communities are surrounded by heavy transportation facilities (e.g., freeways) and activities (e.g., warehousing). While we were aware that PM_2.5_ concentrations were associated with a variety of emission sources, including wildfire events, industrial facilities, and traffic activities, this study aimed to identify the traffic-related component based on the familiarity with the local environments. Firstly, we compared the CAMP PM_2.5_ measurements (from June to December) to the annual PM_2.5_ concentrations from the CalEnviroScreen 4.0 of the 22 households to check the spatial patterns of our ~one-month measurements and the annual concentrations. Secondly, we used the Pearson correlation to explore how traffic-related air pollution is associated with the ambient measurements, and we compared a traditional traffic measurement proxy (i.e., distance to nearest highway calculated from the US Census Bureau) as well as the diesel PM emissions (extracted from the CalEnviroScreen 4.0) with the CAMP PM_2.5_ measurements; diesel PM represents exhaust from on-road (e.g., trucks, buses) and off-road (e.g., trains, ships) sources with diesel engines. Additionally, we ranked the 22 households by the CAMP PM_2.5_ concentrations and the diesel PM emissions separately. We calculated delta rank (CAMP rank minus diesel rank) to assess how the diesel emissions were aligned with the ambient PM_2.5_ measurements. These analyses allowed for exploring air pollution exposure in our targeted disadvantaged communities beyond citizen-science-based monitoring campaigns, reflecting effective strategies to resolve exposure disparities.

## 3. Results and Discussion

Our local hourly correction equation for the PurpleAir measurements is y = 0.78x − 0.10rh + 7.6 (R^2^: 0.87); x represents raw hourly PurpleAir measurements, y represents adjusted hourly PurpleAir measurements, and rh represents relative humidity (see Figure S1). Our QA/QC procedure resulted in 18,034 h of ambient PM_2.5_ concentrations across the 22 households in the study area from June to December 2021. The average PM_2.5_ concentrations during the entire monitoring period were 18.8 µg/m^3^ (see Table S2). Below, we present the major findings of the spatial and temporal patterns of PM_2.5_ exposure as well as the relationship between traffic-related air pollution emission and the ambient PM_2.5_ concentrations.

### 3.1. Spatial Patterns

**Figure 2:**
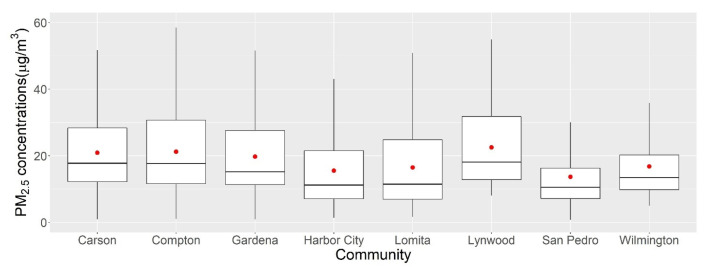
Boxplots of hourly PM_2.5_ concentrations during summer and fall 2021 by community. The red dots indicate average concentrations. The boxes show the interquartile range (IQR). The upper whisker extends from the hinge to the largest value at most 1.5*IQR, while the lower whisker extends from the hinge to the smallest value at most 1.5*IQR.

One of our goals was to evaluate the intra-urban variations in PM_2.5_ exposure in the study area. Figure 2 shows the distribution and statistics of hourly PM_2.5_ concentrations during the two monitoring seasons by community. Lynwood (north of our study area) showed the highest average concentrations and larger variations compared with the lowest values in San Pedro (south of our study area); this result is presumably attributable to the fact that our monitored household in Lynwood was located between westbound I-710 and southbound I-105, two of the major highways in the area. This finding suggests that Lynwood may be heavily impacted by on-road mobile source emissions over time, consistent with the finding of another local study [38]. While San Pedro is located near the Port of LA, a study has found that freeway emissions were the major sources of PM_2.5_ and had much higher emission rates (2–5 times) compared with sources at the Port of LA [39]. This pattern was also consistent with another study reporting lower PM_2.5_ concentrations near the ocean [40]. However, it should be noted that the scarce number (one household in Lynwood and three households in San Pedro) and different surrounding environments (near or away from highway or ocean) of the monitored households may not fully represent community exposure.

**Figure 3:**
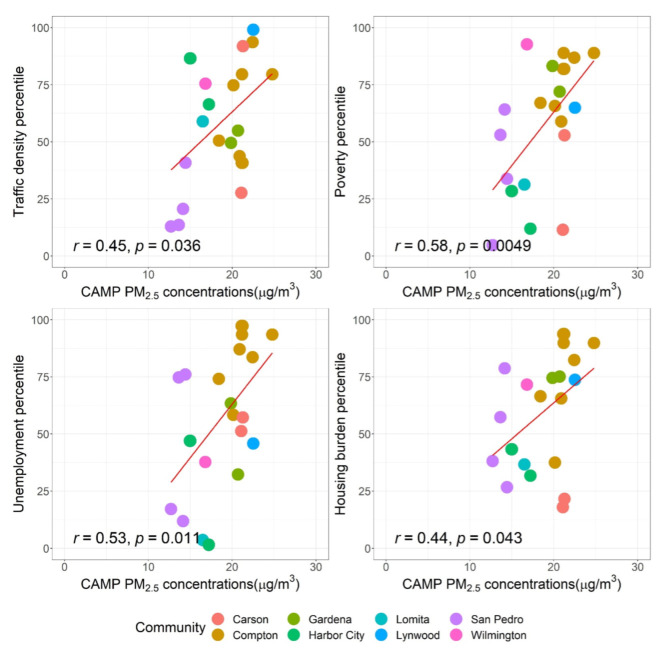
The correlations of residential outdoor PM_2.5_ concentrations and percentiles of traffic density, poverty, unemployment, and housing burden.

We further explored the relationship between the community-level average exposure and the environmental and socioeconomic factors to evaluate the spatial differences. Figure 3 shows the positive correlations of household CAMP PM_2.5_ concentrations and percentiles of traffic density, poverty, unemployment, and housing burden (the Pearson correction coefficient (r): 0.44–0.58). This means communities with denser road networks are more likely to be exposed to higher PM_2.5_ concentrations, consistent with the findings of other studies [41,42,43]. Communities that are burdened by higher rates of unemployment, poverty, and housing tend to be exposed to higher PM_2.5_ concentrations, suggesting potential exposure disparities for disadvantaged communities, as described in other studies [44,45]. Such disparities may be attributable to the disproportionate distribution of PM-emitting facilities [46] and on-road traffic emissions [41].

**Figure 4:**
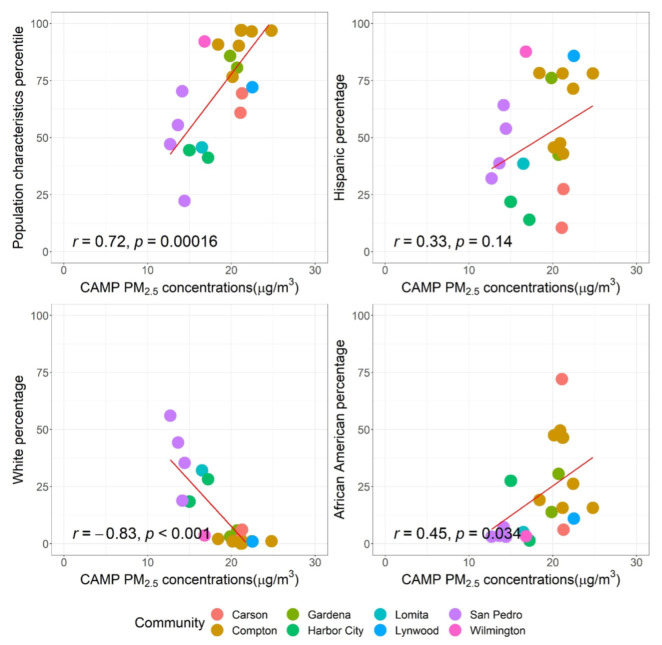
The correlations of residential outdoor PM_2.5_ concentrations and percentiles of population characteristics as well as the percentage of Hispanic, White, and African American population. Population characteristics represent characteristics (e.g., health status, community features) that result in vulnerability to pollution as defined by CalEnviroScreen 4.0.

Similarly, Figure 4 shows the correlations of residential outdoor PM_2.5_ concentrations and percentiles of population characteristics as well as the percentage of Hispanic, White, and African American population. The CAMP PM_2.5_ concentrations showed a strong positive association with percentiles of population characteristics (r: 0.72), and with percentages of Hispanic and African American populations (r: 0.33–0.45), again indicating that communities with a higher people of color population are exposed to poorer air quality [6,8,47,48]. In comparison, the CAMP PM_2.5_ concentrations were negatively associated with the percentage of White population (r: −0.83), providing more evidence regarding air pollution exposure disparities [7,46].

### 3.2. Temporal Patterns

**Figure 5:**
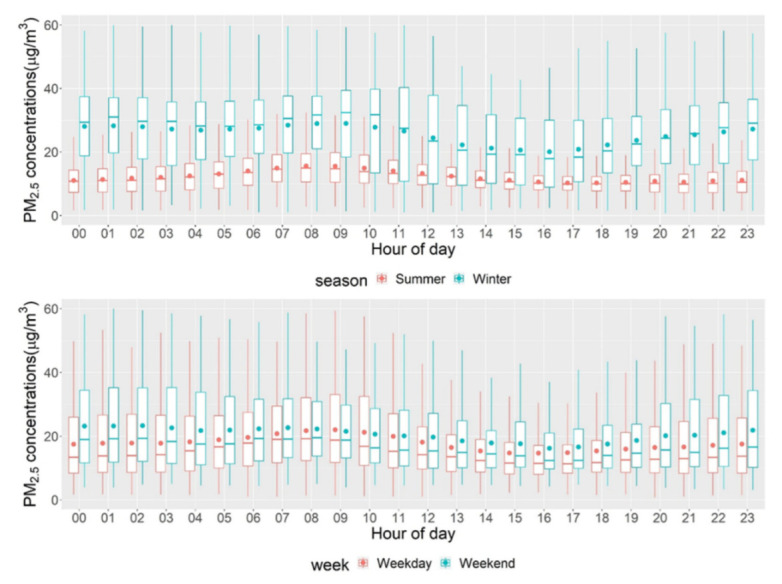
Boxplots of hour-of-day PM_2.5_ concentrations by season and weekday/weekend. The red/blue dots indicate average concentrations. The boxes show the interquartile range (IQR). The upper whisker extends from the hinge to the largest value at most 1.5*IQR, while the lower whisker extends from the hinge to the smallest value at most 1.5*IQR.

We evaluated the temporal variations in the PM_2.5_ exposure of the 22 households. Figure 5 shows the distribution of hourly PM_2.5_ concentrations by season and weekday/weekend. In general, the average CAMP PM_2.5_ concentrations in winter 2021 were much higher compared with the summer concentrations (25.8 µg/m^3^ (winter), 12.4 µg/m^3^ (summer)); August had the highest concentrations during summer, and December had the highest concentrations during winter sampling. This seasonal pattern is consistent with that reported in other studies [49,50]. Particularly, one study conducted in California found that the winter PM_2.5_ could exceed summer PM_2.5_ by 68% [51]. This outcome is perhaps explained by elevated aerosol layers that were not detected by ground-level monitors. Temperature inversion during summer/winter could lead to higher PM_2.5_ concentrations [51]. Additionally, increasing wildfire magnitude and intensity from October to December in Southern California (with extreme seasonal winds such as Santa Ana winds) could contribute to the higher PM_2.5_ concentrations [23].

The average PM_2.5_ concentrations during weekdays were lower than those during weekends (17.9 µg/m^3^ (weekday) vs. 21.0 µg/m^3^ (weekend); see Figure S2), consistent with the results of some studies [50,52,53]. Particularly, Sunday had the highest average PM_2.5_ concentrations, while Tuesday had the lowest (21.8 µg/m^3^ (Sunday) vs. 16.5 µg/m^3^ (Tuesday); see Figure S3). However, other studies identified higher PM_2.5_ concentrations during weekdays [54,55]. A possible explanation may be that there were more travels over weekends during the COVID-19 pandemic across this area.

In terms of the hour-of-day patterns, generally, 9:00 am and 11:00 pm had peak PM_2.5_ concentrations and variations, while 4:00 pm had off-peak concentrations (see Figure S4). However, the between-hour PM_2.5_ data were much smoother than the within-hour data. This variation further reflects the spatial and temporal variations between each community (see Figure S5). For example, Lynwood had higher hourly PM_2.5_ concentrations compared with other communities; the peak hours were around noon. In comparison, the peak hours in Wilmington were around morning time. These outcomes are likely because residents from these disadvantaged communities may have different working shifts with greater car ownership and usage than the regional average. Additionally, disadvantaged communities are often in proximity to warehouses with high volumes of truck traffic, especially during nighttime [56,57]. Other outdoor activities during weekends or nighttime (e.g., barbecue, lawn mowing) may be important anthropogenic sources contributing to high PM_2.5_ concentrations. Overall, our study area may represent valuable temporal patterns among disadvantaged communities and communities in proximity to traffic segments and ports, which warrants further studies.

### 3.3. Temporal Patterns

**Figure 6:**
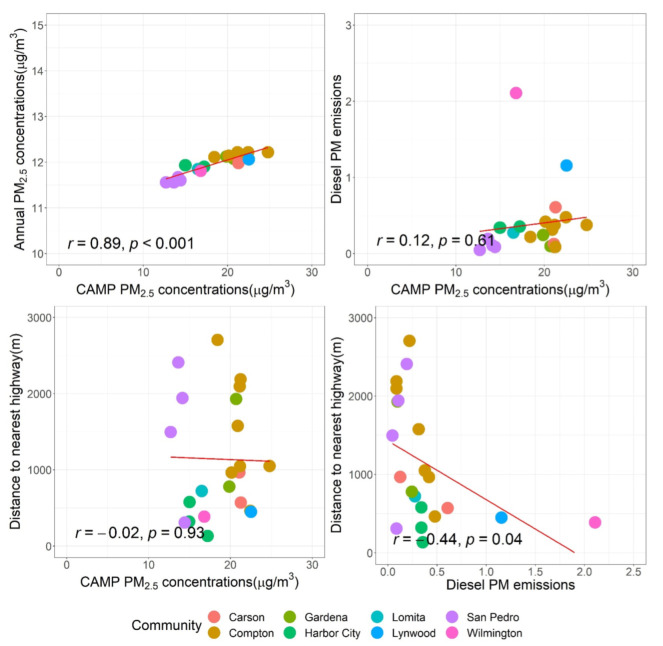
The comparisons of CAMP PM_2.5_ concentrations, annual PM_2.5_ concentrations, distance to nearest highway, and diesel PM emissions by community.

We tested how our CAMP PM_2.5_ measurements were aligned with the annual PM_2.5_ concentrations, traffic-related proxy, as well as diesel emissions from CalEnviroScreen 4.0 (see Figure 6). Overall, the CAMP measurements were consistent with the annual concentrations (r: 0.89), indicating that our ~one-month measurements during two seasons were representative of the annual patterns, which agrees with one of the exposure assessment studies—the European Study of Cohorts for Air Pollution Effects (ESCAPE) [58,59]. Importantly, this finding suggests that air quality monitoring campaigns could be strategically designed to capture the annual patterns, especially for resource-constrained communities.

We found a weak correlation between the CAMP PM_2.5_ measurements and the diesel PM emissions (r: 0.12). This is an indication that traffic emissions are not the only sources for our study area; studies on the source apportionment of urban PM_2.5_ are needed to interpret the source profiles [60]. Additionally, more campaigns targeting traffic-related air pollution (e.g., black carbon; a marker for traffic particles) are needed to improve our understanding of traffic-related emissions in disadvantaged communities. Similarly, we found no correlation between the CAMP PM_2.5_ measurements and distance to the nearest highway (r: −0.02). However, there was a weak, negative correlation between distance to the nearest highway and the diesel PM emissions (r: −0.44). While distance to the nearest highway has commonly been used as a proxy for ambient PM_2.5_ [61] or traffic-related pollutants in particular [62,63], this was not obviously identified in our study, indicating that it may not well represent the traffic-related air pollution concentrations in our context. Therefore, additional proxies (e.g., traffic counts, road density) as well as more traffic-related air pollution monitoring campaigns are recommended to better characterize air pollution exposure across disadvantaged communities.

**Figure 7:**
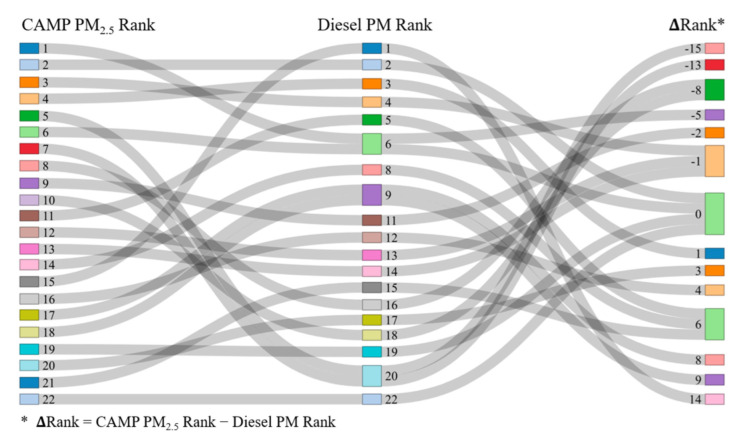
The rank difference between the CAMP PM_2.5_ rank and the diesel PM rank of the 22 households. Each line represents one household. Households ranking at the top were exposed to higher ambient PM_2.5_ or diesel PM emissions; households ranking at the bottom were exposed to lower ambient PM_2.5_ or diesel PM emissions.

We further developed a Sankey plot to explore how the ranks of the CAMP PM_2.5_ concentrations and diesel PM emissions of the 22 households differed (see Figure 7). In general, most households had different ranks between ambient concentrations and diesel emissions, indicating that these households may be exposed to additional sources (e.g., passenger vehicles, industrial facilities) and are impacted by various emission reduction factors and air pollution transport processes [64]. Positive delta rank values suggest that some emission reduction and transport processes were more likely to alleviate the ambient PM_2.5_ exposure, while negative delta rank values suggest that additional emission sources might contribute to the ambient PM_2.5_ concentrations of the households.

These scenarios of delta rank values may have been reflected by the surrounding environments. For example, for a household with a delta rank value of 14 (in Wilmington), the nearest distance to the park (330 m) and sea (800 m) may explain the low ambient PM_2.5_ rank through emission absorbing and reduction, while the nearest distance to highway (350 m) suggests large volumes of trucks that are associated with heavy diesel PM emissions. In comparison, for a household with a delta rank value of −15 (in Compton), the nearest distance to the airport (320 m) possibly explains the additional impacts of these emission sources. Other households are surrounded by a microscale built environment, including a complex of land uses of industrial, residential, and commercial, as well as open spaces and traffic facilities and intersections, which could contribute to their nearby ambient PM_2.5_ concentrations [65,66].

### 3.4. Implications for Exposure Assessment across Disadvantaged Communities

Developing reliable air monitoring campaigns is an important goal for tracking air pollution exposure. There are a limited number of studies that leverages low-cost sensors and citizen science to conduct air pollution monitoring campaigns across disadvantaged communities. Even fewer studies assess spatial and temporal patterns to inform the best exposure assessment practices for these communities. Our citizen-science-based low-cost monitoring campaign identified different PM_2.5_ concentrations and variations across the monitored communities, suggesting that the spatial heterogeneity of PM_2.5_ exposure could be better characterized within the dense networks of low-cost sensors [44,65]. We identified multiple positive/negative correlations between residential outdoor PM_2.5_ concentrations and socioeconomic status, as well as the surrounding built environment. With a limited number of air monitors, the choice of monitoring locations should be strategically considered to capture environments with various land uses, traffic volumes, and population characteristics with the goal of identifying exposure disparities. Our study further justifies the need to expand the low-cost sensor network across disadvantaged communities with the help of community members [10,12,47,53].

Our study showed temporal patterns that draw more attention to disadvantaged communities. We identified much higher average PM_2.5_ concentrations during the winter than summer of 2021, suggesting potential impacts of destructive wildfire activities and meteorological conditions in California [23,51]. The different day-of-week and hour-of-day PM_2.5_ concentration patterns compared with the results of previous studies indicate that these disadvantaged communities may reveal different working shifts, outdoor activities, and exposure to traffic emissions among residents. We also found that our one-month monitoring during summer and winter could be representative of the annual patterns of PM_2.5_. Additional monitoring is needed to evaluate the recommended number of days and seasons of monitoring for assessing long-term air pollution exposure across disadvantaged communities. Our approach of leveraging citizen science and low-cost sensors has the advantage of exploring these nuances for disadvantaged communities. For example, residents’ self-reported activities (e.g., commute pattern) and observations (e.g., wildfire smoke, traffic volumes) along with the low-cost sensor measurement would offer insights into future exposure assessment strategies.

### 3.5. Implications for Healthy Community Development across Disadvantaged Communities

Developing healthy communities while promoting environmental justice has gained momentum over the past few decades. A key question for urban planners is how to design cities and communities in a way that all populations would be exposed to relatively low levels of air pollution. We identified a rank difference between ambient PM_2.5_ concentrations and PM diesel emissions across disadvantaged communities. This finding suggests that the built environment can be targeted to reduce the exposure disparity. For example, a number of studies have found that air pollution exposure could be impacted by small-scale differences in the built environment, including traffic and road geometry [67], impervious surface [68,69], greenness [70], walkability [71], the fragmentation of urban patches [70], and other factors [19,72,73,74]. Oftentimes, disadvantaged communities are near highways, have much fewer green spaces, much greater auto dependence, poor quality pavement streets, and residential segregation patterns [14,46,48], which may be associated with adverse air pollution exposure [73] and disparities [75]. Without increased air monitoring networks among disadvantaged communities, one cannot explore such mechanisms, thus failing to eliminate built environment disparities [76], conduct air pollution exposure assessment [77], inform emission control strategies [43], and assess public health outcomes [6].

### 3.6. Limitations and Implications for the Development of Future Research 

A limitation of our CAMP study is that only 22 participants were recruited due to a limited number of monitors, and all the measurements were collected in residential areas across the disadvantaged communities using fixed-site monitoring. An understudied topic is how the exposure assessment would be improved by the number of monitors, types of surrounding land uses and road hierarchy, and time periods of monitoring. One solution to enhance the monitoring coverage and density for disadvantaged communities is to leverage emerging satellite-based monitoring and mobile monitoring strategies [11,78,79]. Our study only measured ambient air pollution outside of each household. Future monitoring campaigns may consider monitoring both outdoor and indoor air pollution concentrations across disadvantaged communities [53], given that Southern California is very vulnerable to wildfire smoke, and older homes of disadvantaged communities tend to be leakier, with increased infiltration [80,81]. While our study shed light on the relationship between ambient air pollution concentrations and traffic-related emissions, we did not monitor pollutants that are traffic-related (e.g., black carbon) in particular. We also did not delve into source contributions to PM_2.5_ concentrations [82]. Future studies can explore how citizen science and low-cost sensors can be used to assess exposure to traffic-related pollutants and the source apportionment. We did not distinguish any human behaviors (e.g., types of activities, means of transportation) among different racial-ethnic groups and different wind speeds and directions across different communities. Future research could disentangle the spatial and temporal nuances of the exposure based on these factors. Another emerging challenge is how to interpret the collected measurement data and share timely information with community participants. We offered a household report to each participant in the CAMP study, but it will not be enough without a sustainable approach to democratize citizens with reliable technologies and motivation to evaluate their environmental health risks in the long run [26]. Overall, it is a concerted, ongoing effort to engage community participants, university researchers, and environmental agencies to communicate science with residents in a timely and scientific manner for improving exposure assessment and promoting environmental justice.

## 4. Conclusions

We leveraged citizen science and low-cost sensors to develop a community-based air monitoring program outside of 22 households across disadvantaged communities in Southern California from June 2021 to December 2021. We found that communities with a higher percentage of Hispanic and African American population and higher rates of unemployment, poverty, and housing burden were more likely to be exposed to higher PM_2.5_ concentrations. We also identified unique temporal (e.g., hour-of-day, day-of-week) patterns among disadvantaged communities that warrants further studies. The difference between the ambient PM_2.5_ concentrations and diesel PM emissions suggests that the built environment can be targeted to reduce the exposure disparity. The use of low-cost sensors with the participation of community residents offers great potential to effectively assess air pollution exposure and capture “hotspots” across space and time among disadvantaged communities. Our work could be expanded to inform emission control strategies and urban planning policies, thus improving exposure assessment, facilitating epidemiological studies, and promoting environmental justice.

## Data Availability

The data presented in this study are available upon request from the corresponding author.

## Supplementary Materials

The following supporting information can be downloaded at: https://www.mdpi.com/article/10.3390/ijerph19148777/s1, Table S1: Important variables retrieved from the CalEnviroScreen 4.0; Table S2: Summary statistics of the CAMP PM_2.5_ concentrations during the monitoring periods; Figure S1: PurpleAir hourly correction equation using collocated monitors in the City of Compton; Figure S2: Boxplots of hourly PM_2.5_ concentrations by weekday vs. weekend; Figure S3: Boxplots of hour-of-day PM_2.5_ concentrations by day of week; Figure S4: Boxplots of hour-of-day PM_2.5_ concentrations; Figure S5: Boxplots of hour-of-day PM_2.5_ concentrations by community.
