# Supplementary material for: Leveraging Citizen Science and Low-Cost Sensors to Characterize Air Pollution Exposure of Disadvantaged Communities in Southern California
Source: Int J Environ Res Public Health. 2022 Jul 19;19(14):8777. doi: 10.3390/ijerph19148777 (PMC9322770; doi:10.3390/ijerph19148777)
Supplement: Supplementary file 1 [file ijerph-19-08777-s001.zip › ijerph-1811919-supplementary.pdf]

Supplemental Materials

Table S1. Important variables retrieved from the CalEnviroScreen 4.0.

Variable Name	Description
Traffic density percentile	Percentile of traffic density in vehicle-kilometers/hour/road length (within 150m of the census tract)
Poverty percentile	Percentile of percent of population living below two times of the federal poverty level
Unemployment percentile	Percentile of percent of population over 16-year-old that is unemployed and eligible for the labor force
Housing burden percentile	Percentile of percent of housing-burdened low-income households
Population characteristics percentile	Percentile of population characteristics variable scaled with a range of 0-10
Hispanic percentage	2019 ACS population estimates of the percent identifying as Hispanic or Latino
White percentage	2019 ACS population estimates of the percent identifying as White
African American percentage	2019 ACS population estimates of the percent identifying as non-Hispanic African American or Black
$PM_{2.5}$	Annual mean $PM_{2.5}$ concentrations
Diesel PM	Diesel PM emissions from on-road (e.g., trucks, buses) and non-road sources (e.g., trains, ships)

Table S2. Summary statistics of the CAMP $PM_{2.5}$ concentrations during the monitoring periods.

Month (2021)	N	Mean	Min	Q1	Median	Q3	Max	SD
June	2,953	8.4	1.5	5.3	7.3	10.2	33.1	4.7
July	4,393	13.9	3.4	10.3	12.7	15.5	279.5	9.4
August	2,085	14.9	5.0	10.7	14.1	18.0	69.0	5.6
October	456	21.8	4.9	16.5	21.9	26.6	45.6	7.7
November	5,749	24.9	0.7	13.0	24.8	35.4	122.7	13.2
December	2,398	29.0	1.0	19.1	30.7	37.9	190.5	14.0
All periods	18,034	18.8	0.7	9.5	14.7	26.5	279.5	12.8

Note: the unit of the Mean, Min (Minimum), Q1 (Quantile 1), Median, Q3 (Quantile 3), Max (Maximum), and SD (Standard Deviation) is $\mu g/m^3$.

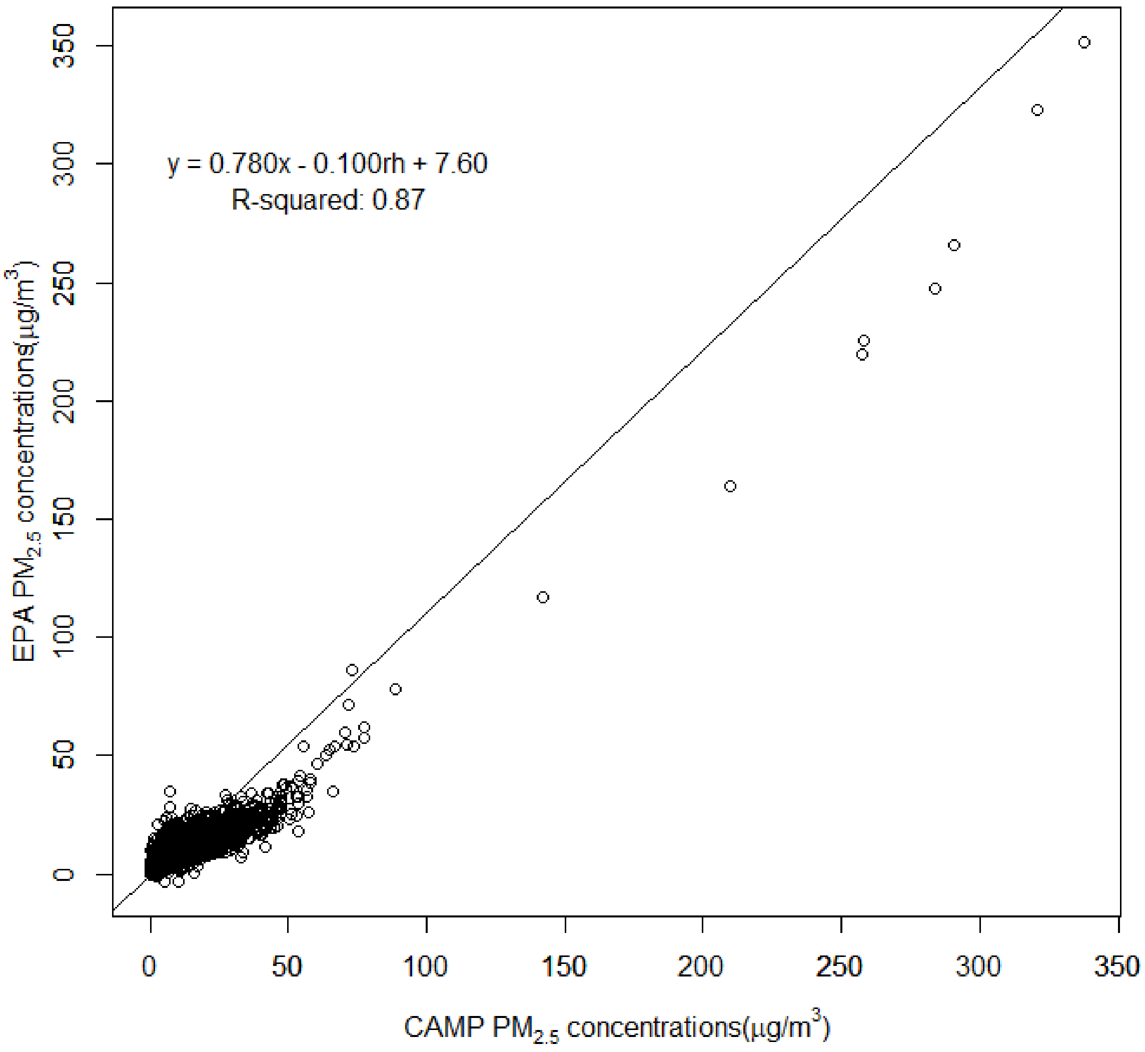

Figure S1. PurpleAir hourly correction equation using collocated monitors in the City of Compton.

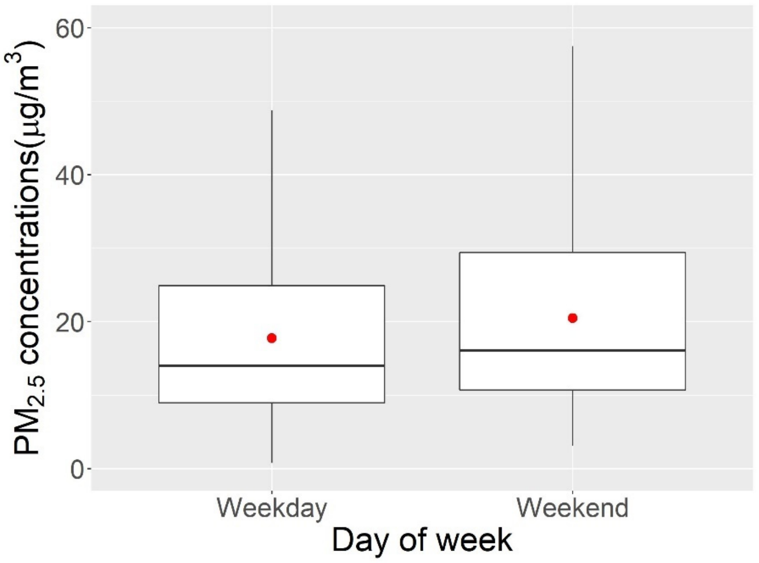

Figure S2. Boxplots of hourly $PM_{2.5}$ concentrations by weekday vs. weekend. The red dots indicate average concentrations. The boxes show the interquartile range (IQR). The upper whisker extends from the hinge to the largest value at most 1.5*IQR while the lower whisker extends from the hinge to the smallest value at most 1.5*IQR.

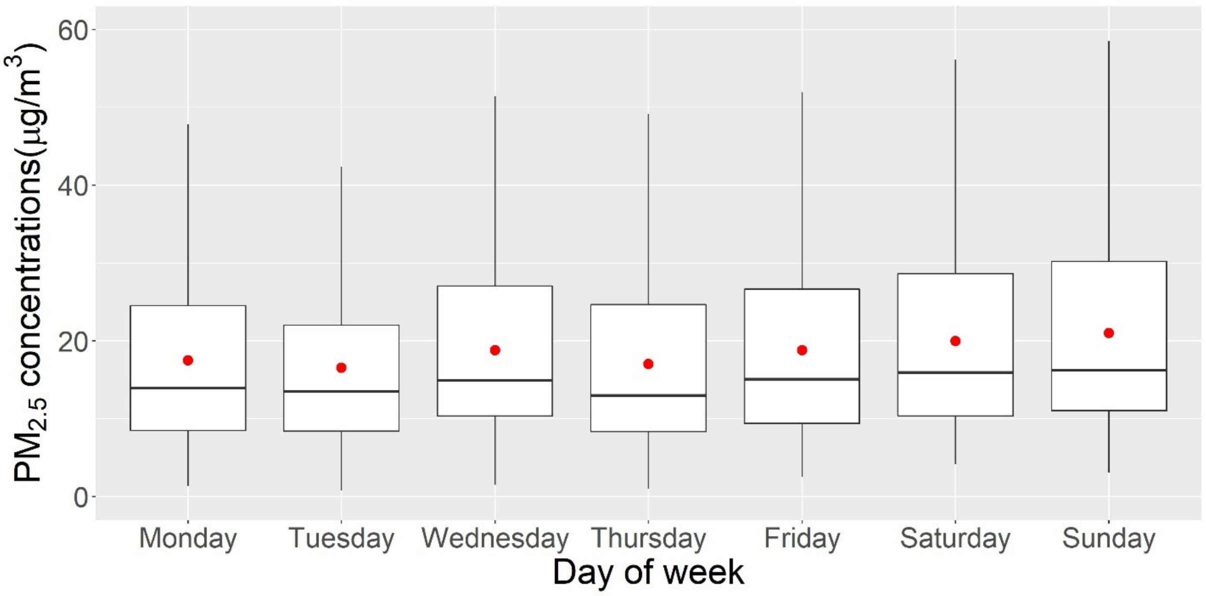

Figure S3. Boxplots of hour-of-day $PM_{2.5}$ concentrations by day of week. The red dots indicate average concentrations. The boxes show the interquartile range (IQR). The upper whisker extends from the hinge to the largest value at most 1.5*IQR while the lower whisker extends from the hinge to the smallest value at most 1.5*IQR.

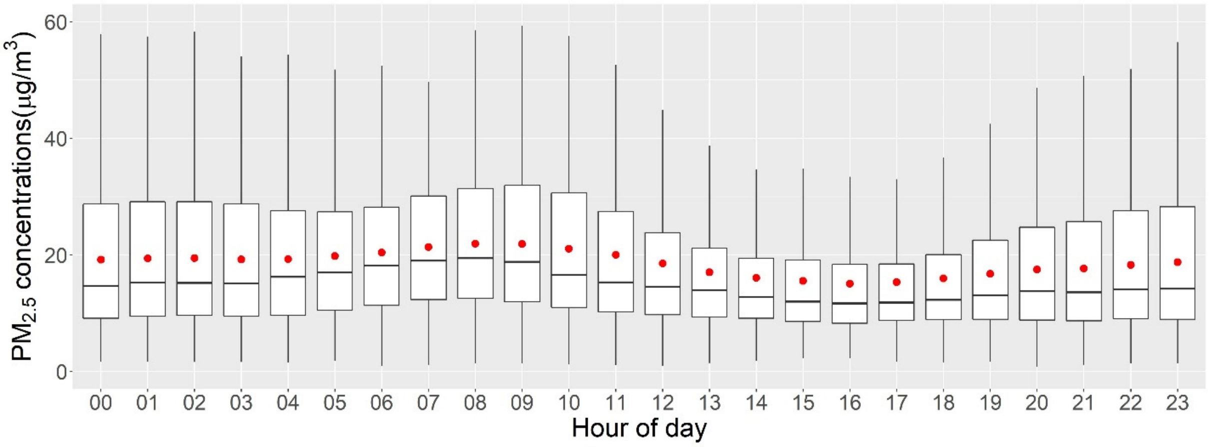

Figure S4. Boxplots of hour-of-day $PM_{2.5}$ concentrations. The red dots indicate average concentrations. The boxes show the interquartile range (IQR). The upper whisker extends from the hinge to the largest value at most 1.5*IQR while the lower whisker extends from the hinge to the smallest value at most 1.5*IQR.

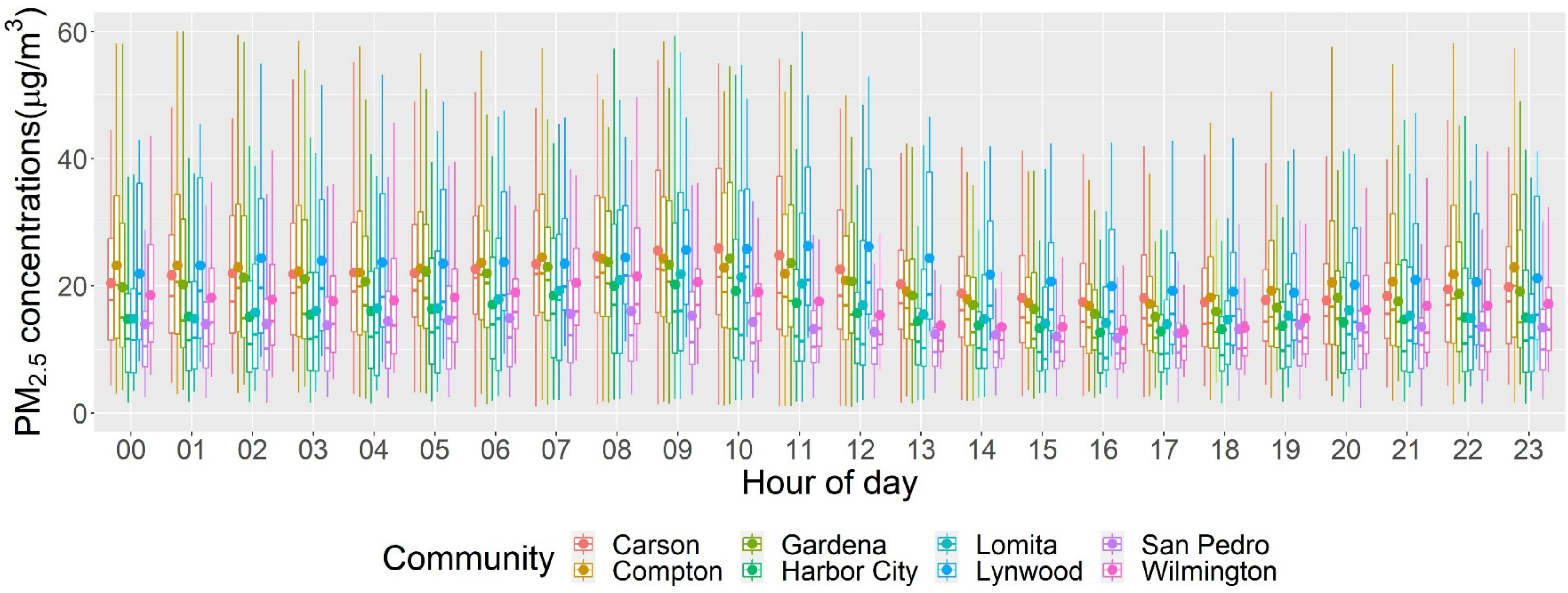

Figure S5. Boxplots of hour-of-day $PM_{2.5}$ concentrations by community. The dots with various colors indicate average concentrations. The boxes show the interquartile range (IQR). The upper whisker extends from the hinge to the largest value at most 1.5*IQR while the lower whisker extends from the hinge to the smallest value at most 1.5*IQR.
